# Mangiferin Ameliorates Cisplatin Induced Acute Kidney Injury by Upregulating Nrf-2 via the Activation of PI3K and Exhibits Synergistic Anticancer Activity With Cisplatin

**DOI:** 10.3389/fphar.2018.00638

**Published:** 2018-06-18

**Authors:** Pritam Sadhukhan, Sukanya Saha, Sayanta Dutta, Parames C. Sil

**Affiliations:** Division of Molecular Medicine, Bose Institute, Kolkata, India

**Keywords:** cisplatin induced nephrotoxicity, oxidative stress, apoptosis, inflammation, antioxidant, anti-inflammatory, anticancer, mangiferin

## Abstract

Occurrence of oxidative stress is the principal cause of acute kidney injury induced by cisplatin. Mangiferin, a naturally occurring antioxidant molecule, is found to ameliorate several oxidative stress mediated pathophysiological conditions including cancer. Cisplatin induced cytotoxicity was measured in NKE cells by MTT assay and microscopic analysis. Induction of oxidative stress and regulation of proapoptotic molecules were subsequently investigated by using different spectrophotometric analyses, FACS and immunocytochemistry. Induction of nephrotoxicity was determined by analyzing different serum biomarkers and histological parameters *in vivo* using swiss albino mice. Activation of NF-κB mediated pro-inflammatory and caspase dependent signaling cascades were investigated by semi-quantitative RT-PCR and immunoblotting. Mangiferin was found to ameliorate cisplatin induced nephrotoxicity *in vitro* and *in vivo* by attenuating the induction of oxidative stress and upregulating Nrf-2 mediated pro-survival signaling cascades via the activation of PI3K. Additionally, mangiferin showed synergistic anticancer activity with cisplatin in cancer cell lines (MCF-7 and SKRC-45) and EAC cell induced solid tumor bearing experimental mice. The ameliorative effect of mangiferin is primarily attributed to its anti-oxidant and anti-inflammatory properties. It acts differentially in normal tissue cells and tumor cells by modulating different cell survival regulatory signaling molecules. For the first time, the study reveals a mechanistic basis of mangiferin action against cisplatin induced nephrotoxicity. Since Mangiferin shows synergistic anticancer activity with cisplatin, it can be considered as a promising drug candidate, to be used in combination with cisplatin.

## Introduction

Cisplatin, *cis*-diamminedichloroplatinum (II), is a widely used anticancer drug and is effective against several types of cancers in almost all parts of the body, including cancers of the breast, lung, ovary, testis, head, and neck. Cisplatin was first identified as an inhibitor of cell cycle in 1965 and in due course of time its therapeutic efficacy has been studied widely (Sadhukhan et al., 2016; Alves de Souza et al., 2017). Despite its chemotherapeutic activity, different studies reported the toxicity of this molecule in several vital organs of the human body including heart, liver, brain, spleen, and with the most significant deleterious effect in renal tissues (Fatima et al., 2016). However, cisplatin remains to be the only drug option for several forms of cancers till date. The cisplatin induced nephrotoxicity was first reported 30 years ago and thereafter several studies have been undertaken to elucidate the molecular mechanism underlying cisplatin induced diseased condition in the renal tissue (Kodama et al., 2014; Potocnjak and Domitrovic, 2016). These mechanistic approaches are still not fully studied and therefore require further investigations. Renal dysfunction or nephrotoxicity due to the administration of cisplatin can be classified in many ways and among them, acute kidney injury (AKI) is found to occur in more than 30% patients (Huang et al., 2017). AKI is one of the common and severe pathophysiological states associated with drug toxicity, shock, ischemia-reperfusion, sepsis, etc. and can be categorized by the rapid decline in the kidney function and extensive tubular damage. It has a very high mortality rate and is reported to cause multiple organ damages (Succar et al., 2017). Different reports suggest that elevated level of inflammatory cytokines and intracellular reactive oxygen species (ROS) mediated mechanisms are involved with the AKI associated cytotoxicity (Burmeister et al., 2017). Elevation of intracellular ROS in the kidney, leads to the occurrence of oxidative stress and induction of several inflammatory signaling cascades through the activation of nuclear factor-κB (NF-κB) (Kumar et al., 2015; Huang et al., 2017). Different natural products studied in the last decade are reported to have potential anti-inflammatory and antioxidant activity against different oxidative stress mediated organ pathophysiology (Baradaran et al., 2015). Extensive research has been done with these bioactive molecules to identify complementary and alternative medicines (Manna et al., 2007; Das et al., 2009; Chowdhury et al., 2016; Ghosh et al., 2016; Sarkar et al., 2016). These molecules have been found to be effective against several pathophysiological conditions and interestingly these natural molecules show no significant harmful effects in dietary doses. Therefore, studying the effects of different natural compounds against cisplatin induced kidney dysfunction is perceived to be significant (Sarkar and Sil, 2006; Pal et al., 2011; Tamadon et al., 2014; Yang et al., 2016a).

Plant derived bioactive compounds are preferred to the chemically synthesized drugs because of their pleotropic and non-toxic nature (Agarwal et al., 2015; Dutta et al., 2017; Rashid et al., 2017). Different classes of molecules have so far been identified from the diversified plant environment but there is still a lot to be revealed. Among the ones identified, flavonoids are the most abundant group of bioactive compounds found in the plant kingdom and are also common in human diets (Saha et al., 2016b). In the flavonoid family, mangiferin, a non-steroidal polyhydroxy polyphenolic molecule, found predominantly in the bark of the *Mangifera indica*, has pleotropic activity (Pal et al., 2013, 2014). It has free radical scavenging activity and can inhibit as well as ameliorate ROS induced oxidative damage against several pathophysiological conditions (Ghosh et al., 2012; Matkowski et al., 2013; Song et al., 2017). Previous studies indicated that pretreatment of renal cells with mangiferin prevents oxidative damage induced by tert-Butyl hydroperoxide (tBHP) via modulating the PI3K-AKT mediated signaling pathway and activation of Nrf-2 (Saha et al., 2016c). Several reports also suggest that mangiferin has anti-inflammatory activity and can modulate various cytokine-induced signal transduction events thereby improving metabolic disorders like diabetes, cardiovascular diseases etc (Pal et al., 2014). Further, by inhibiting neurological disorders (like depression, sedation and neurodegenerative disorders and increasing nervous sensation) mangiferin has also proven itself as an effective neuroprotector (Yang et al., 2016b; Wang et al., 2017). Till date, the effect of mangiferin against cisplatin induced nephrotoxicity has not been studied. Based on those previous findings, we hypothesized that supplementing of mangiferin with cisplatin would be a novel strategy to protect the kidney from cisplatin induced oxidative damages.

The present study is initiated with an aim to evaluate the protective effect of mangiferin against nephrotoxicity following cisplatin administration in normal human kidney epithelial cells (NKE cells) and in swiss albino mice. Our results showed that mangiferin can significantly inhibit the induction of oxidative stress by cisplatin in NKE cells and cellular death. Furthermore, histopathological examinations showed that in swiss albino mice, mangiferin attenuates the functional and structural damages in kidney, induced by cisplatin. Mangiferin is found to suppress both the elevation of intracellular ROS and activation of the several pro-inflammatory cytokines as well as NF-κB. This molecule also diminished the renal cell apoptosis through the modulation of different apoptotic proteins, p53 and Nrf-2 related signaling cascades. Since mangiferin also possesses significant anticancer activity, we have also evaluated the anti-cancer effect of cisplatin in presence of mangiferin, both *in vitro* and *in vivo*. Overall, these findings indicate the possible attenuative role of mangiferin against cisplatin induced nephrotoxicity and its synergism with cisplatin’s anti-cancer effect.

## Materials and Methods

### Chemicals

Mangiferin, Cisplatin, H_2_-DCFDA, JC-1 and FITC conjugated Annexin V apoptosis detection kit was purchased from Sigma-Aldrich Chemical Company (St. Louis, MO, United States). Dulbecco’s Modified Eagle’s Medium (DMEM), MEM and other chemicals like antibiotics, trypsin etc. was purchased from HIMEDIA (Mumbai, India). Fetal bovine serum (FBS) was bought from HyClone (Thermo Scientific Hy-Clone, Logan, UT, United States).

The primers used for RT-PCR were purchased from IDT. The antibodies used were purchased from Abcam (Cambridge, United Kingdom), Cell Signaling Technology (Danvers, MA 01923, United States), BioBharati Lifescience (India). 3-(4,5-dimethylthiazol-2-yl)-2,5-diphenyltetrazolium bromide (MTT) along with all other necessary chemicals and reagents were of analytical grade and purchased from the SISCO Research Laboratory, Mumbai, India.

### *In Vitro* Model of Cisplatin Induced Renal Injury

The normal kidney epithelial (NKE) cell line was obtained from Cleveland Clinic Foundation, United States. This renal cell was derived from the uninvolved kidney tissue of a patient with renal cell carcinoma. The cells were immortalized by transduction of the human telomerase subunit. NKE cells were maintained in RPMI medium supplemented with 10% Fetal bovine serum (FBS) and antibiotics at 37°C in culture flasks with 5% CO_2_. Confluent monolayers (80%) of NKE cells were subjected to exposure of cisplatin, mangiferin and other molecules as per the experimental design. LC50 dose of cisplatin on NKE cells was determined in this study and was used for all the experiments.

### Determination of Dose and Time Dependent Effect of Cisplatin and Mangiferin in Renal Cells

Dose and time dependent toxicity of cisplatin on the NKE cells were quantified using MTT cell viability assay. The experiments were performed as described elsewhere (Saha et al., 2016c). Briefly, to determine the dose dependent toxicity, the cells were seeded on a 96 well culture plate at a density of 5 × 10^4^ cells per well in 100 μl serum supplemented culture media. After overnight incubation, the cells were exposed to cisplatin at a dose of 2, 5, 10, 15, 20, 25, 30, 40, and 50 μM in a serum free medium. The cells were incubated for 24 h and the media was replaced by 1X PBS containing MTT (0.5 mg/ml). Following an incubation period of 4 h, the MTT crystals (formazon) were dissolved in DMSO and the absorbance was taken using a spectrophotometer at 570 nm.

To determine time dependent cytotoxicity, the NKE cells were exposed to LC50 dose of cisplatin for varied durations (6, 12, 24, and 48 h) in cisplatin containing growth medium. After determining the appropriate dose and time for cisplatin exposure, mangiferin was tested to quantify its protective action. To perform this experiment, the cells were pretreated with mangiferin for 2 h at varied doses ranging from 5 to 30 μM followed by the exposure of cisplatin. Absorbance was subsequently measured at 570 nm.

To further confirm the cytotoxicity and protective action of cisplatin and mangiferin respectively the cells were photographed after incubation of mangiferin and cisplatin at desired dose and time using bright field microscopy at 10X magnification.

### Determination of the Mode of Cell Death

The mode of cell death, *in vitro*, was primarily quantified by the FACS analysis following the protocol as described elsewhere (Sinha et al., 2015). Briefly, after the complete drug exposure, the cells were resuspended in annexin V binding buffer and stained with Annexin V dye for 30 min. Next, the cells were washed in 1X PBS and analyzed by BD FACS Calibur Flow Cytometry System at an excitation of 485 nm and emission of 530 and 590 nm. The mode of cell death was further confirmed by fluorescent microscopy by using DAPI staining following the protocol described elsewhere (Saadat et al., 2015). Briefly, the cells were grown on a 6 well culture dish with a glass cover slip coated with poly L-lysine. After the complete drug exposure, the cells were washed with 1X PBS and the cover slips were gently mounted over a glass slide with a mounting medium containing DAPI. The slides were dried and finally observed under a fluorescent microscope. For these experiments to quantify the cytotoxic effect of cisplatin, the cells were exposed to 25 μM of cisplatin for 24 h and to enumerate the protective action of mangiferin the cells were pretreated with 20 μM of mangiferin for 2 h prior to cisplatin exposure.

### Determination of the Involvement of Oxidative Stress

To investigate the effect of cisplatin induced cytotoxicity in the intracellular ROS level, *in vitro*, the following experiments were performed. First of all, intracellular ROS level was quantified by the FACS analysis using the H_2_-DCFDA staining. The analysis was done following the protocol described elsewhere (Saha et al., 2016a). Briefly, after the complete drug exposure the cells were stained with 5 mM H_2_-DCFDA dye for 30 min. Next, the cells were washed in 1X PBS and analyzed by BD FACS Calibur Flow Cytometry System at an excitation of 485 nm and emission of 530 and 590 nm. To reconfirm the involvement of oxidative stress in cisplatin induced cytotoxicity and its attenuation by mangiferin pretreatment, the cells were also pretreated with 5 mM NAC (*N*-acetyl cysteine) prior to cisplatin exposure. The consequences of oxidative stress were further analyzed by estimating the level of lipid peroxidation and protein carbonylation using thiobarbituric acid (TBA) and 2,4-Dinitrophenylhydrazine (DNPH) respectively. The activity of major antioxidant enzymes such as superoxide dismutase (SOD), catalase (CAT), glutathione S-transferase (GST), glutathione reductase (GR), glutathione peroxidase (GPx), were measured following the protocol described elsewhere (Manna et al., 2008a). Reduced glutathione (GSH) and oxidized glutathione (GSSG) were measured according to protocol as described elsewhere (Dutta et al., 2017). Briefly, SOD activity was determined by mixing 5 μg of protein with sodium pyrophosphate buffer, PMT and NBT. The reaction was initiated by adding NADH to the reaction mixture. It was incubated for 90 s at 30°C. The reaction was stopped with the addition of 1 ml glacial acetic acid and finally the absorbance was measured at 590 nm. CAT activity was determined by mixing 5 μg of protein with H_2_O_2_. The reaction was continued for 10 min at 25°C. The absorbance of the solution was recorded at 240 nm. GST activity was determined by mixing 25 μg of protein with potassium dihydrogen phosphate (KH_2_PO_4_), ethylenediaminetetraacetic acid (EDTA), 1-chloro-2,4-dinitrobenzene (CDNB) and GSH. The temperature was maintained at 37°C and the absorbance was recorded at 340 nm for 5 min. GR activity was determined my mixing 50 μl of protein with KH_2_PO_4_, EDTA, 5,5′-dithiobis(2-nitrobenzoic acid) (DTNB) and NADPH, GSSG and water. The change in absorbance of the reaction mixture was recorded at 412 nm at 37°C for 3 min. GPx activity was determined by mixing 50 μl of sample with KH_2_PO_4_, EDTA, NaN3, GR and GSH. The mixture was incubated for 10 min at 37°C and NADPH was added. The reaction was started with the addition of H_2_O_2_ and finally the change in the absorbance was recorded at 340 nm for 5 min. GSH content was determined by mixing the sample with DTNB and the absorbance was recorded at 412 nm. The GSSG content in the sample was determined by mixing the sample with *N*-ethylmaleimide (NEM) to prevent the formation of GSH from GSSG. The sample was then mixed with disodium hydrogen phosphate (Na_2_HPO_4_) and DTNB. Finally the absorbance was measured at 412 nm.

### Determination of the Mitochondrial Membrane Potential (MMP)

To investigate the effect of cisplatin induced cytotoxicity on the MMP, FACS analysis was performed by rhodamine 123 staining. Briefly, after complete drug exposure, the cells were stained with 5 mM rhodamine 123 dye for 30 min. Next, the cells were washed in 1X PBS and analyzed by BD FACS Calibur Flow Cytometry System at an excitation of 485 nm and emission of 530 and 590 nm (Pal et al., 2014). The change in mitochondrial membrane potential was further confirmed by fluorescent microscopy experiments using JC-1 staining (Morigi et al., 2015).

### Determination of the Activity of Cleaved Caspase-3

NKE cells were cultured on glass coverslips and were subjected to cisplatin and mangiferin exposure as described above. The immunochemistry analysis was performed according to the protocol as described elsewhere (Das et al., 2015). The cells were incubated with the anti-active caspase 3 antibody for overnight at 4°C followed by anti-rabbit IgG (FITC conjugated) for 2 h. The slides were analyzed using fluorescent microscope (Olympus BX61). The activity of cleaved caspase 3 was further validated by incubating the cells with 50 μM Z-VAD-FMK (a pan caspase inhibitor) prior to cisplatin exposure, and cell viability was measured by performing MTT assay according to the protocol described above.

### *In Vivo* Model of Cisplatin Induced Acute Renal Injury and Its Amelioration by Mangiferin Administration

Four weeks old male swiss albino mice were used for this study. The animals were obtained from Central Animal house and research facility of Bose Institute, Kolkata, India. All the animals were acclimatized for 2 weeks in an alternating 12 h light/dark cycles and provided with water *ad libitum* and standard diet. Pilot studies were performed to analyze the nephrotoxic potential of cisplatin and ameliorative efficacy of mangiferin in swiss albino mice. For this, different doses of cisplatin (2, 5, 10 mg per kg bw weekly once for 21 days) and mangiferin (10, 20, 40 mg per kg bw in alternative days for 21 days) were used and after analyzing the experimental results, the nephrotoxic model was developed.

Briefly, the experimental design for this study was as follows:

Animals were divided into four groups (six animals/group).
  Group 1 (Control): Animals received vehicles (Olive oil used for mangiferin treatment) only.
  Group 2 (Mag treated): Animals received mangiferin orally at a dose of 20 mg/kg bw on alternative days for 21 days.
  Group 3 (Cis treated): Animals received cisplatin intraperitoneally at a dose of 10 mg/kg bw weekly once for 21 days.
  Group 4 (Cis + Mag treated): Animals received cisplatin interperitoneally at a dose of 10 mg/kg bw weekly once and mangiferin 20 mg/kg bw on alternative days for 21 days.

At the end of the experimental procedure, the mice were fasted for 12 h and blood was rapidly collected by cardiac puncture. The kidneys were then carefully dissected out and directly snap frozen in liquid nitrogen and stored at -80°C.

All the animal experiments were performed according to the guidelines of the Institutional Animal Ethical Committee (IAEC), Bose Institute, Kolkata [IAEC/BI/3(I) cert. /2010] and full details of the work plan with experimental animals were approved by IAEC as well as CPCSEA (Committee for the Purpose of Control and Supervision on Experiments on Animals), Ministry of Environment and Forests, New Delhi, India (1796/PO/Ere/S/14/CPCSEA).

### Histological Examination

For histopathological examinations, kidney tissues were isolated from different experimental animals and were fixed in formalin buffer (10%) and embedded in paraffin. Approximately 6 μm sections were made and mounted on glass slides. The slides were then stained by using hematoxylin and eosin. The slides were examined in a magnification of 400X under a light microscope.

### Estimation of the Blood Urea Nitrogen (BUN) and Creatinine Level

Estimation of BUN facilitates the assessment of acute kidney injury, indicating the renal health. Like BUN, serum creatinine is also another major indicator of the renal health. Creatinine is produced by metabolism in the muscle and is removed from the blood mainly by glomerular filtration. BUN and creatinine were quantified in blood serum, collected from each animal after the entire experimental protocol. The experiments were performed following the protocol as described in the kits (Span Diagnostic Ltd., India) (Pal et al., 2015b).

### Determination of the Involvement of Oxidative Stress in the Renal Tissue

To investigate the involvement of oxidative stress in the renal tissue following the cisplatin and mangiferin administration, intracellular ROS, lipid peroxidation and protein carbonylation were quantified according to the protocol as described earlier in the Section “Determination of the Involvement of Oxidative Stress.”

### Estimation of the Intracellular Antioxidant Enzymes and Metabolites

To further confirm the occurrence of oxidative stress in the renal tissue due to cisplatin exposure and its amelioration by mangiferin administration, different intracellular antioxidant enzymes (SOD, CAT, GST, GR and GPx) were measured spectrophotometrically in the renal tissue homogenates of all the experimental animals. The experiments were performed according to the protocol as described earlier in the Section “Determination of the Involvement of Oxidative Stress” (Manna et al., 2007).

### Myeloperoxidase Activity (MPO) Assay

The MPO activity was measured to evaluate the infiltration of neutrophils in the renal tissue due to cisplatin administration. The MPO activity in the tissue homogenate was measured according to the protocol as described elsewhere (Sahu et al., 2014). Briefly, 100 mg of the tissue was homogenized in 50 mM phosphate buffer (pH 6, 4°C) containing 0.5% hexadecyltrimethylammonium bromide. The resulting homogenate was then subjected to freeze-thaw cycles for three times and was finally centrifuged for 10 min at 30000 rpm, 4°C. MPO activity was measured by quantifying the peroxide dependent oxidation of *o*-dianisidine. The reaction mixture was then incubated at 37°C for 10 min. Finally, the spectrophotometric absorbance was noted at 460 nm.

### Isolation of the Renal Mitochondria and Estimation of the Several Mitochondrial Enzyme Activities

Mitochondria were isolated from the renal tissue following the protocol as described elsewhere. Briefly, 300 mg of the tissue was homogenized in an isolation buffer (10 mM Tris, 250 mM Sucrose, 1mM EGTA, pH-7.2) at 4°C. The homogenate was then centrifuged at 600 ×*g* for 10 min and the supernatant was further centrifuged at 10,000 ×*g* for 10 min to obtain the mitochondrial pellet. The pellet was then washed in the isolation buffer with and without EGTA for twice followed by centrifugation as above. Finally, the mitochondrial protein fraction was stored in a buffer containing 10 mM Tris and 250 mM sucrose. The protein content was then quantified using the BCA reagent following the manufacturer’s protocol (Sinha et al., 2015).

#### NADH Dehydrogenase (NDH) Activity Assay

The activity of NDH was measured spectrophotometrically according to the protocol as described elsewhere (Sahu et al., 2014). Briefly, 100 μg of mitochondrial protein was added to a mixture containing 0.2 M glycylglycine (pH8.5), 6 mM NADH in 2 mM glycylglycine buffer and 10.5 mM cytochrome C. The O.D. was measured at 550 nm and the change was recorded for 3 min at an interval of 15 s. NDH activity was expressed as percentage of the control group.

#### Succinate Dehydrogenase (SDH) Activity Assay

The activity of SDH was measured spectrophotometrically according to the protocol as described elsewhere (Sahu et al., 2014). Briefly 100 μg of mitochondrial protein was mixed to a mixture containing 0.2 M phosphate buffer (pH 7.8), 1% BSA, 0.6 M succinic acid and 0.03 M potassium ferricyanide. The O.D. was measured at 420 nm and the change was recorded for 2 min at an interval of 15 s. SDH activity was expressed as percentage of the control group.

#### Mitochondrial Redox Activity Assay

Mitochondrial redox activity was spectrophotometrically measured using the MTT dye following the protocol as described earlier. Briefly, 100 μl of the mitochondrial samples were incubated with the MTT solution for 4 h at 37°C. The formazan crystals were then dissolved with DMSO and the O.D. reading was taken at 590 nm. It was expressed as percentage of the control group.

### Reverse Transcriptase Assay for the Determination of the Cytokines Level

Total RNA was isolated from the mice renal samples using the TRIzol reagent, according to the standard protocol (Invitrogen). Following this, the RNA concentration was quantified spectrophotometrically. Two μg of RNA from each sample was converted to cDNA using cDNA synthesis kit, following which PCR was performed. The PCR products were then subjected to electrophoresis on 1.5% agarose gel (**Table 1**).

**Table 1:** The exact product size and annealing temperatures of the primers.

Gene	Primer sequence (5′ TO 3′)	Annealing temp (C)	Amplicon size (bp)
β actin	FP: ACATTGGCATGGCTTTGTTT	53.8	193
	RP: GTCCTCAGCCACATTTGTAG		
IL 1β	FP: GAGTGTGGATCCCAAGCAA	50.1	174
	RP: TCCTGACCACTGTTGTTTCC		
TNFα	FP: TCTCAGAATGAGGCTGGATAA	55.0	188
	RP: CCCGGCCTTCCAAATAAATAC		
IL-6	FP: GATAAGCTGGAGTCACAGAAG	58.7	163
	RP: TTCTGACCACAGTGAGGAATG		
IL-10	FP: CACTGAGCTTCTCTGTGAACTA	50.5	191
	RP: GTGGCCAGCCTTAGAATAGAA		

### Western Blotting

Cytosolic and nuclear proteins were isolated from the tissue homogenate following the protocol of Sinha et al. (2015). For western blot analysis, equal amount of protein samples (50 μg in each well) were subjected to gel electrophoresis using 10–12% sodium dodecyl sulfate-polyacrylamide gel (SDS-PAGE). The proteins in the gel were transferred to the activated PVDF membranes and the membranes were first blocked using 3% BSA for 1 h. Then the membranes were incubated overnight at 4°C with a buffer containing respective primary antibodies NF-κB, rabbit monoclonal 1:1000, Cell Signaling Technology; Bax (rabbit monoclonal 1:1000, Cell Signaling Technology), Bcl-2 (rabbit monoclonal 1:1000, Cell Signaling Technology), cytochrome C (rabbit monoclonal 1:1000, Cell Signaling Technology), cleaved caspase-3 (rabbit monoclonal 1:1000, Cell Signaling Technology), calpain (rabbit monoclonal 1:1000, Cell Signaling Technology), cleaved caspase 12 (rabbit monoclonal 1:1000, Cell Signaling Technology), Nrf-2 (rabbit monoclonal 1:1000, Cell Signaling Technology), HO-1 (rabbit monoclonal 1:1000, Cell Signaling Technology) and SOD-2 (rabbit monoclonal 1:1000, Cell Signaling Technology). β-actin was used as the loading control for the cytosolic proteins and Lamin B1 for the nuclear proteins. Followed by this, the primary antibodies were detected against HRP-conjugated secondary antibody using the HRP substrate ECL solution.

### Determination of the Anti-tumor Efficacy of Cisplatin

To investigate the therapeutic efficacy of cisplatin in presence of mangiferin, different *in vitro* experiments on SKRC-45 (metastatic human renal cell carcinoma cell line) and MCF-7 (human breast cancer cells) cells were performed. The cells were exposed with both mangiferin and cisplatin simultaneously in a dose dependent manner. Cytotoxicity was then quantified using MTT cell viability assay and bright field microscopy [performed according to the protocol as described earlier in the Section “Determination of Dose and Time Dependent Effect of Cisplatin and Mangiferin in Renal Cells”].

Further, to determine the anti-tumor efficacy of cisplatin under mangiferin administered experimental animals, Ehrlich ascites carcinoma (EAC) solid tumor model in swiss albino mice were developed following the protocols as described elsewhere (Kim et al., 2016). EAC cells were subcutaneously implanted in the right flank of the 6 weeks old male swiss albino mice. After 10 days, the animals were randomly divided into four groups containing 4 animals in each. The groups were as follows: Control (untreated tumor bearing mice), Mag treated (tumor bearing mice treated with mangiferin at a dose of 20 mg/kg bw in alternative days for 21 days), Cis treated (tumor bearing mice treated with cisplatin at a dose of 10 mg/kg bw weekly once and mangiferin 20 mg/kg bw on alternative days for 21 days), Mag and cis treated (tumor bearing mice treated with both mangiferin and cisplatin for 21 days) (**Figure 14A**). For all the experimental animals, cisplatin was administered intraperitoneally and mangiferin orally. After the complete experimental period, the tumors were dissected out and the volumes were quantified using a Vernier caliper using the ellipsoid volume equation. Simultaneously tumor weights were also measured.

### Statistical Analysis

Statistical analysis was performed using the Origin, version 8.0 software. The same software was used to determine the mean value of a given parameter among the various experimental groups by performing the one-way analysis of variance (ANOVA) with the Tukey test. All the experimental data were represented as the mean ± standard deviation (mean ± SD) or as percent activity compared to the control group. A *p*-value equal to or less than 0.05 were considered as statistically significant.

## Results

### Dose Dependent Effect of Cisplatin and Mangiferin, *in Vitro*

**FIGURE 1:**
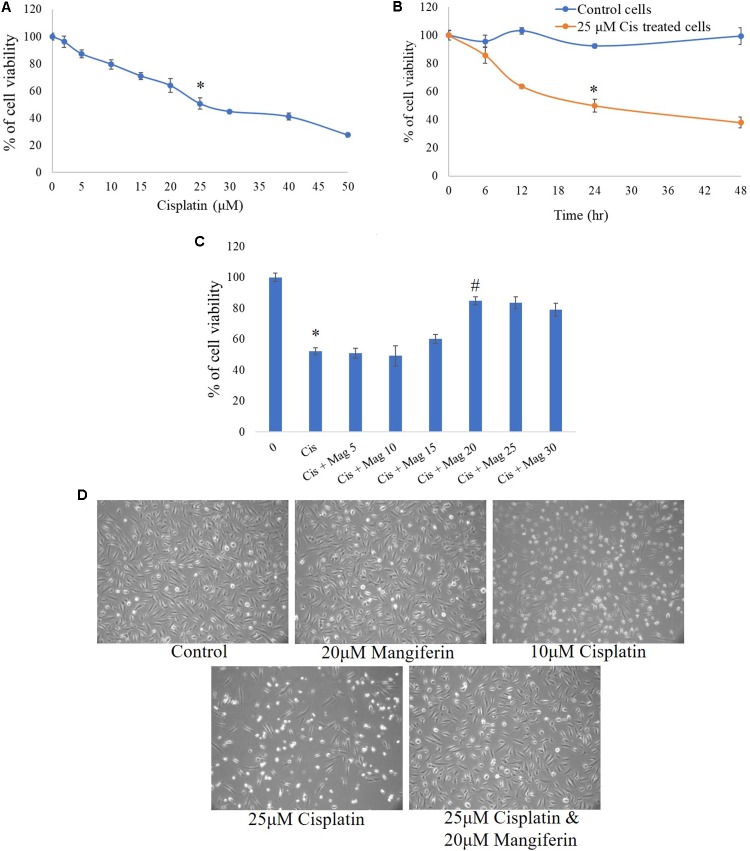
MTT cell viability assay was performed to estimate the cytotoxic effect of cisplatin and its amelioration by pre-exposure of mangiferin. **(A)** Dose dependent effect of cisplatin on NKE cells at varied doses (0–50 μM). **(B)** Time dependent cytotoxic effects of 25 μM cisplatin on NKE cells at varied time intervals (0–48 h). **(C)** Ameliorative effect of mangiferin at varied doses (5–30 μM) against 25 μM cisplatin exposure for 24 h. Each column represents mean ± SD, *n* = 3. “^∗^” represents the significant difference with the control cells (^∗^*P* < 0.05). “#” represents significant difference with the cisplatin exposed cells (^#^*P*< 0.05). **(D)** Microscopic analysis (100X) on the morphology of the NKE cells when exposed to only mangiferin, only cisplatin (10 and 25 μM), both cisplatin and mangiferin.

MTT cell viability assay was performed to determine the cytotoxic effect of cisplatin on NKE cells. It was observed that at 25 μM concentration, the cell viability was 50.7% (SD ± 4%) over control cells (**Figure 1A**). In a time dependent study, it was observed that after 24 h of cisplatin exposure, the cell viability was 50.05% (SD ± 4%) over control cells (**Figure 1B**). In mangiferin pretreated cells (2 h), 20 μM could effectively reverse the cytotoxic effect of cisplatin (**Figure 1C**).

The cytotoxic effect of cisplatin and protective action of mangiferin, as indicated in the MTT cell viability assay, was reconfirmed by using phase contrast microscopy. Exposure to 25 μM cisplatin for 24 h could effectively change the morphology of the cells. The magnitude of cellular damage is observed in a dose dependent manner. However, these alterations in the morphology of the cells were significantly attenuated in the 20 μM mangiferin pretreated cells (**Figure 1D**).

### Effect of Cisplatin and Mangiferin

#### On Apoptosis

**FIGURE 2:**
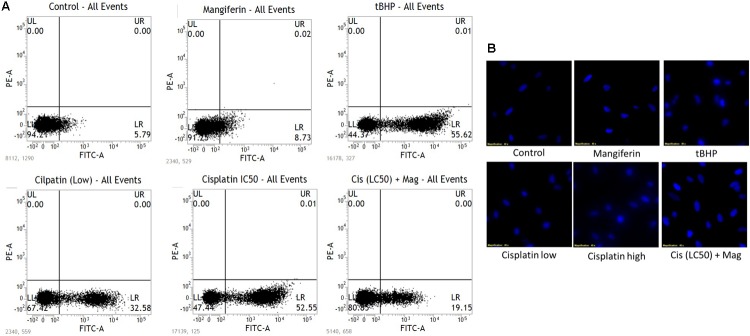
**(A)** FACS analysis with Annexin V staining to determine the percent of apoptotic cells in the different experimental group. **(B)** Fluorescent micrographs (200X) with DAPI stain to observe the effect of cisplatin exposure on DNA fragmentation and its amelioration by mangiferin pre-exposure. Control: untreated cells; Mangiferin: cells were exposed to 20 μM mangiferin; tBHP: Positive control, cells were exposed to 50 μM tBHP; Cisplatin (low): cells were exposed to 10 μM cisplatin; Cisplatin (high): cells were exposed to 25 μM cisplatin; Cis(LC50) + Mag: cells were exposed to 25 μM cisplatin and 20 μM mangiferin. For all the experimental groups the cells were exposed different molecules for 24 h.

To quantitatively measure the induction of apoptosis in the NKE cells after cisplatin exposure, the cells were stained with annexin V after the treatment protocol. It was observed that in 25 μM cisplatin exposed group, 52% cells were annexin V positive. In this experiment tBHP was used as a positive control. On the contrary, in mangiferin pretreated cells the cytotoxic effect of cisplatin was significantly attenuated and only 19.5% of cells were found to be annexin V positive (**Figure 2A**).

The induction of apoptosis was further confirmed by performing fluorescent microscopy with DAPI. The presence of fragmented nuclear material was evident from the micrographs of cisplatin exposed cells (10 and 25 μM). This result clearly indicates the occurrence of apoptosis in the cisplatin exposed cells. However, mangiferin pretreatment was found to be effective in preventing the induction of apoptosis in the cisplatin exposed cells (**Figure 2B**).

#### On Oxidative Stress

**FIGURE 3:**
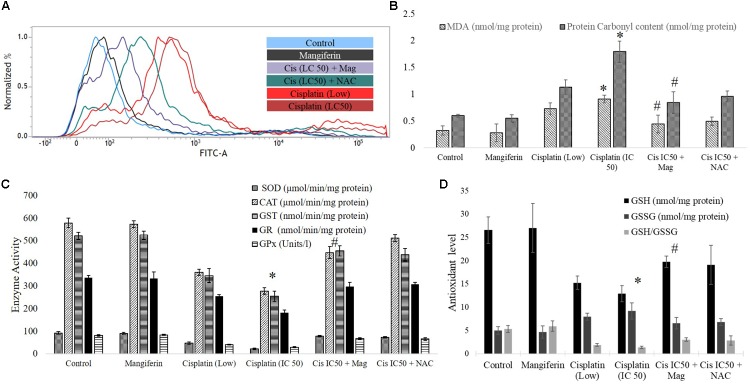
Involvement of oxidative stress in cisplatin induced cytotoxicity and it amelioration by mangiferin pre-exposure in NKE cells. **(A)** FACS analysis using DCFDA stain to quantify the intracellular ROS. **(B)** Quantification of lipid peroxidation and protein carbonylation. **(C)** Quantification of activity of several antioxidative enzymes (SOD, CAT, GST, GR, GPx). **(D)** Quantification of the intracellular GSH and GSSG. Control: untreated cells; Mangiferin: cells were exposed to 20 μM mangiferin; Cisplatin (low): cells were exposed to 10 μM cisplatin; Cisplatin (LC50): cells were exposed to 25 μM cisplatin; Cis(LC50) + Mag: cells were exposed to 25 μM cisplatin and 20 μM mangiferin. Cis(LC50) + NAC: cells were exposed to 25 μM cisplatin and 5 mM NAC. For all the experimental groups the cells were exposed different molecules for 24 h. Each column represents mean ± SD, *n* = 3. “^∗^” Represents the significant difference with the control cells (^∗^*P* < 0.05). “#” represents significant difference with the cisplatin exposed cells (^#^*P*< 0.05).

To investigate the role of oxidative stress in cisplatin induced renal cell injury, different oxidative stress related parameters were measured. It was found that cisplatin can dose dependently elevate the intracellular ROS level in the NKE cells (increase in the intensity of the green fluorescence), which is found to be attenuated by mangiferin pretreatment (**Figure 3A**). Different biomarkers of oxidative stress, i.e., MDA and protein carbonyl contents were also measured in the renal cells, and it was found that cisplatin exposure significantly induced lipid peroxidation and protein carbonylation in the renal cells compared to the control cells (**Figure 3B**). Moreover, cisplatin also dose dependently downregulated the activities of different antioxidant enzymes (SOD, CAT, GST, GR, and GPx) and level of non-enzymatic cellular metabolites (GSH) in the renal cells (**Figures 3C,D**). Further, the level of GSSG was increased dose dependently which led to a decreased GSH/GSSG ratio. For each parameter, mangiferin pretreatment was found to be effective in attenuating the induction of oxidative stress in the renal cells. The antioxidant effect of mangiferin was further validated by using 5 mM NAC. It was found that, like mangiferin, pretreatment of NAC can also attenuate the elevation of ROS and manifestation of oxidative stress in the renal cells.

#### On Mitochondrial Dysfunction

**FIGURE 4:**
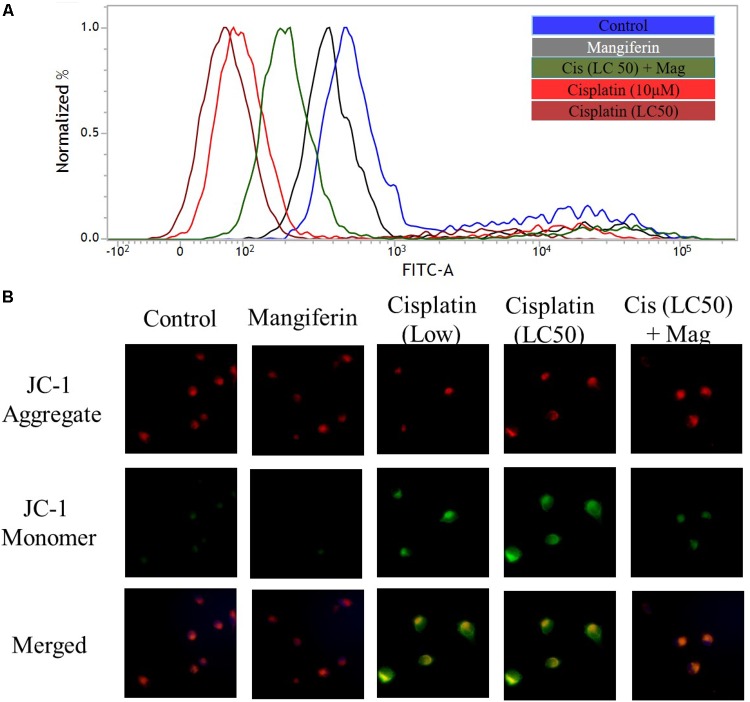
Cisplatin induced mitochondrial dysfunction in NKE cells. **(A)** FACS analysis using rhodamine 123 stain to quantify the MMP. **(B)** Fluorescent microscopic analysis (200X) using JC-1 to investigate the change in MMP in the NKE cells. Control: untreated cells; Mangiferin: cells were exposed to 20 μM mangiferin; Cisplatin (10 μM): cells were exposed to 10 μM cisplatin; Cisplatin (LC50): cells were exposed to 25 μM cisplatin; Cis(LC50) + Mag: cells were exposed to 25 μM cisplatin and 20 μM mangiferin.

To investigate the effect of cisplatin on mitochondrial dysfunction, MMP was measured in the renal cells by using FACS and fluorescent microscopy. FACS analysis with rhodamine 123 dye revealed a significant dose dependent decrease in MMP (decrease in green fluorescence) in cisplatin exposed cells (**Figure 4A**). In microscopic analysis with JC-1 dye, an increasing ratio of green fluorescence and red fluorescence confirmed the decrease in MMP (**Figure 4B**). In both the experiments, it was found that 2 h pre-treatment of 20 μM mangiferin can significantly prevent the loss of MMP due to 25 μM cisplatin exposure for 24 h.

#### On the Expression of Cleaved Caspase-3

**FIGURE 5:**
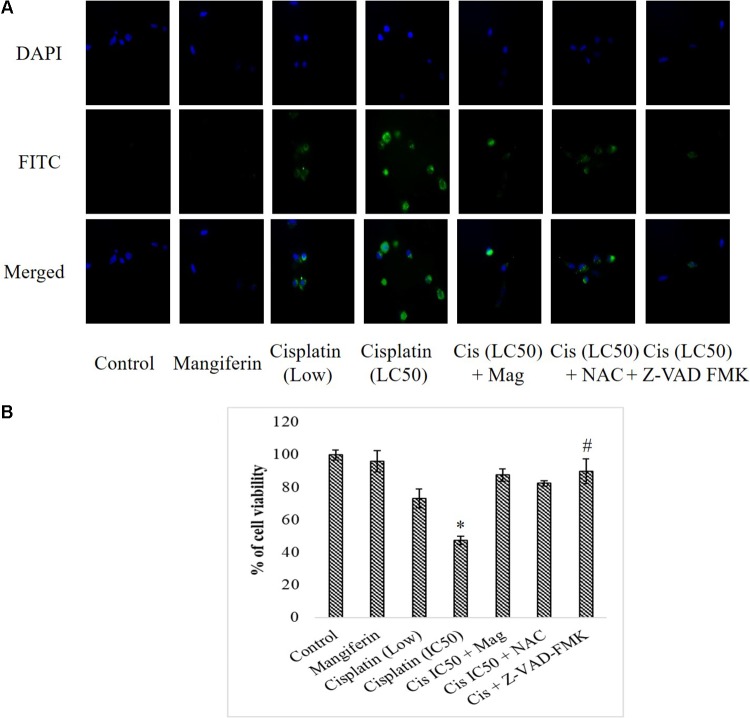
Effect of cisplatin induced cytotoxicity on the intracellular cleaved caspase 3. **(A)** Immunocytochemical analysis to investigate the intracellular expression of cleaved caspase-3. The micrographs were taken under 200X magnification. **(B)** MTT cell viability assay to investigate the effect of caspase inhibition on the cytotoxic effect of cisplatin on NKE cells. Control: untreated cells; Mangiferin: cells were exposed to 20 μM mangiferin; Cisplatin (low): cells were exposed to 10 μM cisplatin; Cisplatin (LC50): cells were exposed to 25 μM cisplatin; Cis(LC50) + Mag: cells were exposed to 25 μM cisplatin and pre-treated with 20 μM mangiferin. Cis(LC50) + NAC: cells were exposed to 25 μM cisplatin and pre-treated with 5 mM NAC. Cis(LC50) + Z-VAD-FMK: cells were exposed to 25 μM cisplatin and pre-treated with 50 μM Z-VAD-FMK. Each column represents mean ± SD, *n* = 3. “^∗^” Represents the significant difference with the control cells (^∗^*P* < 0.05). “#” Represents significant difference with the cisplatin exposed cells (#*P* < 0.05).

To investigate the effect of cisplatin on the intracellular expression of cleaved caspase 3 in NKE cells, fluorescent microscopy was performed. It was found that cisplatin could significantly increase the expression of cleaved caspase 3 in NKE cells in a dose dependent manner. This led to apoptosis in the renal cells. Mangiferin pretreatment prior to cisplatin exposure was found to be effective in attenuating the expression of cleaved caspase 3 (**Figure 5A**). In another experiment, when the renal cells were pre-exposed to Z-VAD-FMK (a pan caspase inhibitor), a sharp decline was observed in the cytotoxic effect of cisplatin (**Figure 5B**).

### Dose Dependent Effect of Cisplatin and Mangiferin, *in Vivo*

**FIGURE 6:**
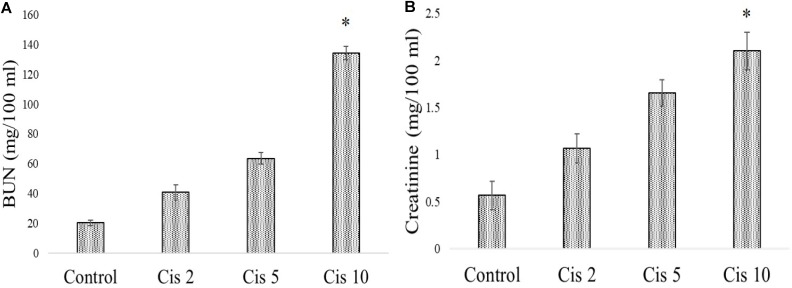
Dose dependent nephrotoxic effects of cisplatin on swiss albino mice. **(A)** Serum BUN **(B)** Serum creatinine levels were measured in experimental animals administered with varied dose of cisplatin (2,5, 10 mg/kg bw). Each column represents mean ± SD, *n* = 6. “^∗^” Represents the significant difference with the vehicle treated animals (^∗^*P* < 0.05). “#” Represents significant difference with the cisplatin administered animals (^#^*P*< 0.05).

In experimental animals, cisplatin administration for 3 weeks with varied doses showed dose dependent toxicity as evident from the dose dependent increase of BUN and creatinine in the serum of the experimental animals. It was observed that the serum BUN and creatinine levels were maximum in the animals administered with 10 mg/kg bw cisplatin compared to the vehicle treated animals and other animals administered with cisplatin at lower doses (2 and 5 mg/kg bw) (**Figures 6A,B**). Hence, 10 mg/kg bw was considered to be an optimum dose to induce nephrotoxicity.

**FIGURE 7:**
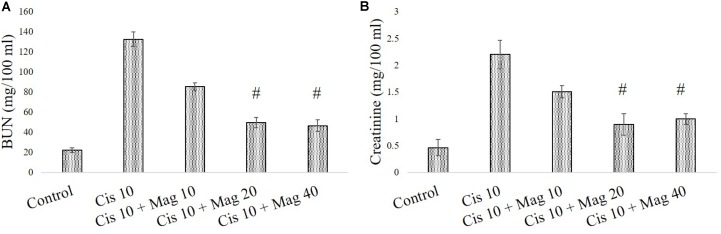
Dose dependent nephroprotective effects of mangiferin on swiss albino mice upon cisplatin exposure. **(A)** Serum BUN **(B)** Serum creatinine levels were measured in experimental animals administered with 10 mg/kg bw of cisplatin and varied dose of mangiferin (5, 10, 20, 40 mg/kg bw). Each column represents mean ± SD, *n* = 6. “^∗^” Represents the significant difference with the vehicle treated animals (^∗^*P* < 0.05). “#” represents significant difference with the cisplatin administered animals (^#^*P*< 0.05).

In a different set of experimental animals, it was found that mangiferin has a significant protective effect against cisplatin induced renal toxicity in a dose dependent manner. Estimation of serum BUN and creatinine level indicated that mangiferin exerts maximum protection at a dose of 20 mg and 40 mg/kg bw compared to the other doses (5 and 10 mg/kg bw) (**Figures 7A,B**). Hence 20 mg/kg bw of mangiferin was used in the subsequent experiments.

### Effect of Mangiferin on Cisplatin Induced AKI

#### Renal Tissue Damage

**FIGURE 8:**
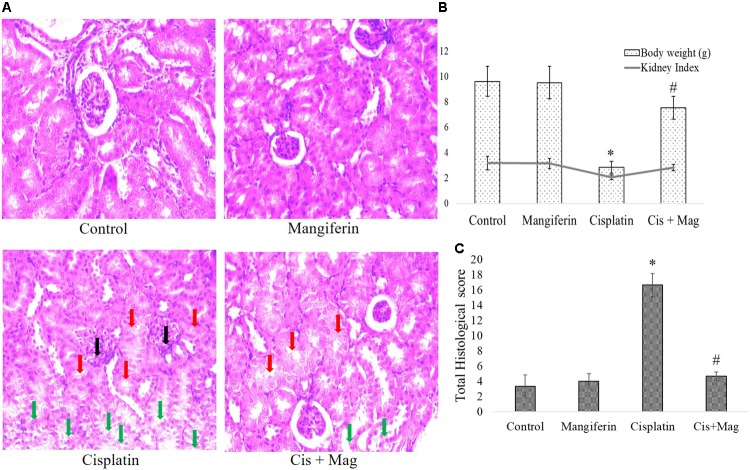
Quantification of renal tissue damage in response to cisplatin administration. **(A)** Microscopic observation of histopathological slides of renal tissue section (400X). Black arrows: the atrophy of a renal corpuscle and glomerulus, Red arrows: the degenerated proximal tubular cells, Green arrows: the damaged distal tubular cells. **(B)** Quantification of histopathological examination. Quantitative assessment of renal injury was represented as number of damaged tubule, renal corpuscles and glomerular atrophy per total cross-sectional area of the renal tissue. **(C)** Estimation of body weight change before and after the experimental procedure and kidney index at the end of protocol. Control: vehicle treated cells. Mangiferin: 20 mg/kg bw mangiferin treated. Cisplatin: 10 mg/kg bw cisplatin treated. Cis + Mag: Treated with both mangiferin and cisplatin. Each column represents mean ± SD, *n* = 6. “^∗^” Represents the significant difference with the vehicle treated animals (^∗^*P* < 0.05). “#” Represents significant difference with the cisplatin administered animals (^#^*P*< 0.05).

In histopathological examination it was clearly observed that mangiferin administration at 20 mg/kg bw could significantly reduce the histopathological changes in the renal tissue induced by 10 mg/kg bw of cisplatin after a period of 3 weeks (**Figure 8A**). In **Figure 8B** histopathological micrographs showed significant tubular damage in renal tissue of animals administered with cisplatin, compared to the control animals. In the micrographs, black arrows indicate the atrophy of a renal corpuscle and glomerulus, the red arrows indicate the degenerated proximal tubular cells and the green ones indicate the damaged distal tubular cells. At the end of the entire experiments, the body weight and kidney weight of animals were noted. The change of body weight in the cisplatin administered animals was significantly less compared to the control animals. The kidney index [(kidney weight/body weight)^∗^100] also showed a significantly decreased value in cisplatin administered animals compared to other experimental groups (**Figure 8C**).

#### Serum Biomarkers

**FIGURE 9:**
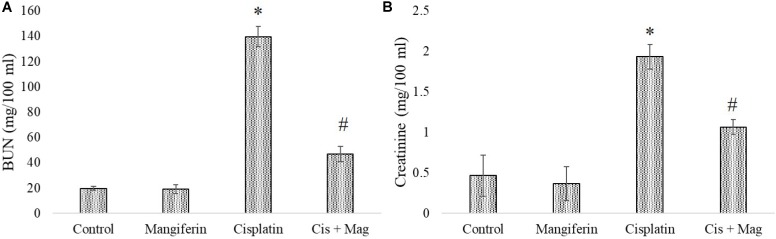
Estimation of stress responsive biomarkers for renal tissue damage in response to cisplatin and mangiferin. **(A)** Serum BUN levels. **(B)** Serum creatinine levels. Control: vehicle treated cells. Mangiferin: 20 mg/kg bw mangiferin treated. Cisplatin: 10 mg/kg bw cisplatin treated. Cis + Mag: Treated with both mangiferin and cisplatin. Each column represents mean ± SD, *n* = 6. “^∗^” Represents the significant difference with the vehicle treated animals (^∗^*P* < 0.05). “#” represents significant difference with the cisplatin administered animals (^#^*P*< 0.05).

As indicated in the pilot experiments, 20 mg/kg bw of mangiferin can significantly ameliorate the level of BUN and creatinine in the blood serum, which was increased due to cisplatin administration at a dose of 10 mg/kg bw weekly once for 3 weeks. It was observed that the BUN and creatinine levels in only cisplatin administered animals are 139.66 mg/dl (SD ± 8.02) and 1.93 ± 0.15 mg/dl respectively. In mangiferin administered cisplatin treated animals the levels of BUN and creatinine were significantly lowered to 47 mg/dl (SD ± 6.91) and 1.06 mg/dl (SD ± 0.09) respectively (**Figures 9A,B**).

#### Oxidative Stress

**FIGURE 10:**
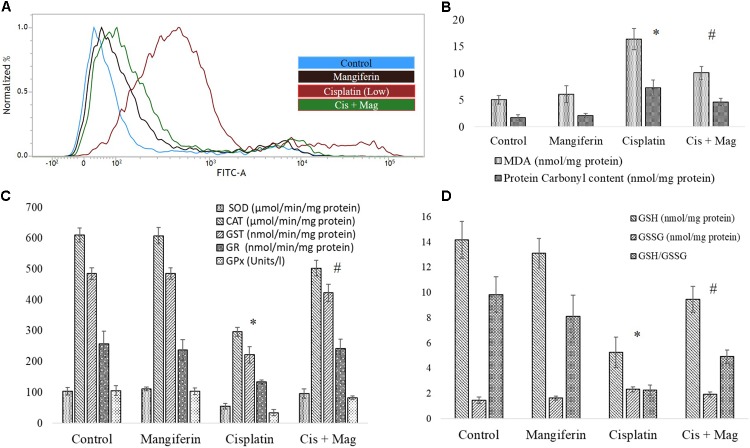
Quantification of oxidative stress induction in cisplatin induced renal damage and its amelioration by mangiferin administration. **(A)** Estimation of ROS by DCFDA staining. **(B)** Quantification of lipid peroxidation and protein carbonylation. **(C)** Quantification of intracellular antioxidant enzyme activity. **(D)** Quantification of intracellular GSH activity. Control: vehicle treated cells. Mangiferin: 20 mg/kg bw mangiferin treated. Cisplatin: 10 mg/kg bw cisplatin treated. Cis + Mag: Treated with both mangiferin and cisplatin. Each column represents mean ± SD, *n* = 6. “^∗^” Represents the significant difference with the vehicle treated animals (^∗^*P* < 0.05). “#” Represents significant difference with the cisplatin administered animals (^#^*P*< 0.05).

FACS analysis revealed that cisplatin administration for 3 weeks could significantly increase the level of ROS in the renal tissue. Simultaneous administration of 20 mg/kg bw mangiferin was found to attenuate the increase of ROS in the experimental animals (**Figure 10A**). It was also found that cisplatin administration could significantly induce lipid peroxidation and protein carbonylation (**Figure 10B**). It has also downregulated the activity of different antioxidant enzymes (SOD, CAT, GST, GR and GPx) (**Figure 10C**) and cellular metabolites (reduced GSH) (**Figure 10D**) compared to the control animals. The altered factors in the cisplatin administered animals were found to be under homeostasis in cisplatin and mangiferin co-exposed animals.

#### Mitochondrial Dysfunction

**FIGURE 11:**
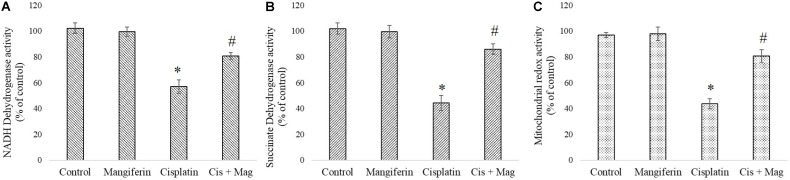
Quantification of mitochondrial dysfunction in cisplatin induced renal damage and its amelioration by mangiferin administration. **(A)** NADH dehydrogenase activity. **(B)** Succinate dehydrogenase activity. **(C)** Mitochondrial redox activity. Control: vehicle treated cells. Mangiferin: 20 mg/kg bw mangiferin treated. Cisplatin: 10 mg/kg bw cisplatin treated. Cis + Mag: Treated with both mangiferin and cisplatin. Each column represents mean ± SD, *n* = 6. “^∗^” Represents the significant difference with the vehicle treated animals (^∗^*P* < 0.05). “#” represents significant difference with the cisplatin administered animals (^#^*P*< 0.05).

Activity of two important mitochondrial enzymes (NDH and SDH) and mitochondrial redox activity were found to be downregulated in cisplatin administered animals compared to the control animals. 20 mg/kg bw mangiferin administration was found to be effective in restoring the activity of mitochondrial redox activity and the two mitochondrial enzymes (**Figure 11**).

#### Renal Inflammation

**FIGURE 12:**
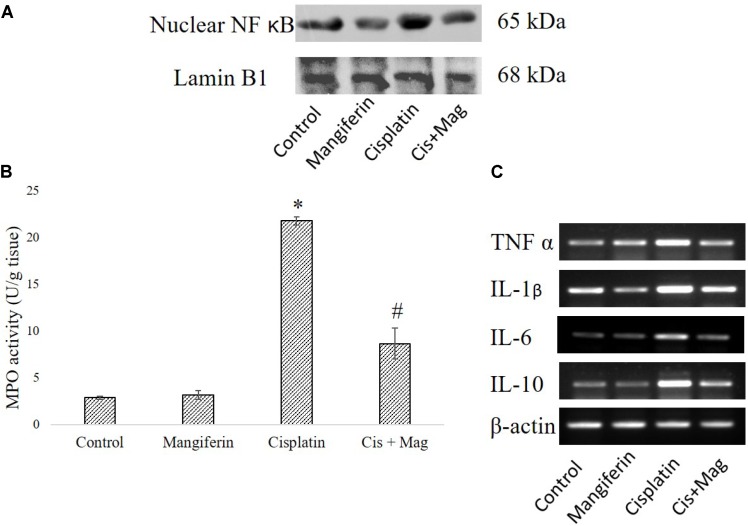
Effect of cisplatin induced renal dysfunction on inflammatory response. **(A)** Expression level of Nuclear NF-κB in renal tissue. **(B)** MPO activity in the renal tissue. **(C)** Semi-quantitative RT-PCR analysis of different inflammation regulatory genes. Control: vehicle treated cells. Mangiferin: 20 mg/kg bw mangiferin treated. Cisplatin: 10 mg/kg bw cisplatin treated. Cis + Mag: Treated with both mangiferin and cisplatin. Each column represents mean ± SD, *n* = 6. “^∗^” Represents the significant difference with the vehicle treated animals (^∗^*P* < 0.05). “#” Represents significant difference with the cisplatin administered animals (^#^*P*< 0.05).

Cisplatin exposure significantly increased the level of NF-κB in the nuclear fraction of the renal tissue of the cisplatin administered animals compared to control animals. Simultaneous administration of mangiferin was found to be effective in restoring the level of NF-κB in the nuclear fraction (**Figure 12A**). Myeloperoxidase activity was significantly increased in the cisplatin treated animals compared to the control animals. Several other proinflammatory cytokines (TNF-α, IL-1β, IL-6, IL-10) were also found to be elevated in the cisplatin administered animals. Mangiferin administration was found to restore the level of MPO and proinflammatory cytokines in cisplatin and mangiferin co-exposed animals (**Figures 12B,C**).

#### Regulation of Different Cell Survival Regulatory Proteins

**FIGURE 13:**
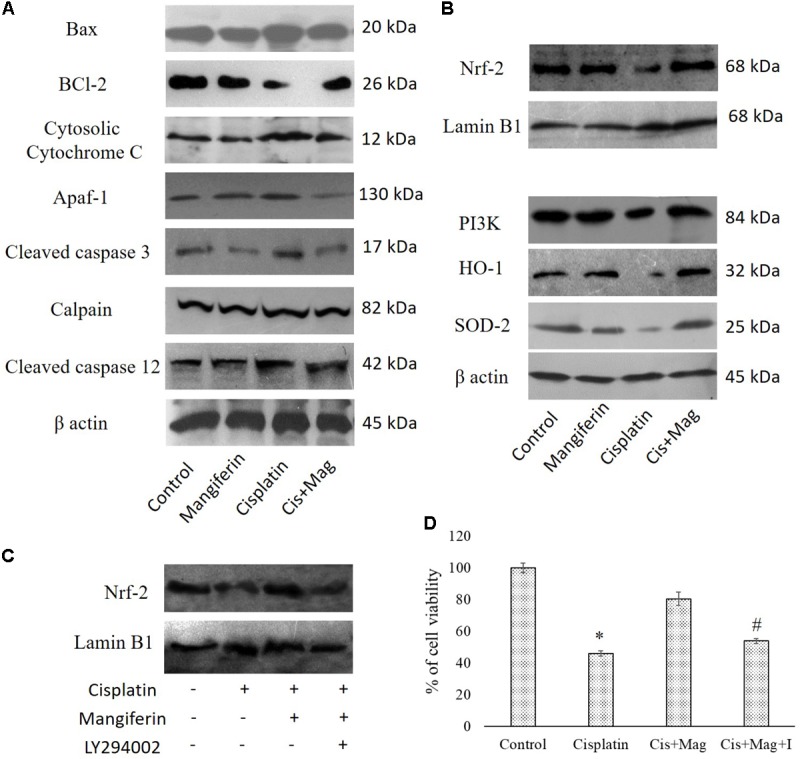
Effect of cisplatin induced renal dysfunction on stress responsive cellular proteins. **(A)** Effect of different stress responsive proapoptotic signaling cascades. **(B)** Effect of different stress responsive cell survival regulatory signaling cascades. Control: vehicle treated cells. Mangiferin: 20 mg/kg bw mangiferin treated. Cisplatin:10 mg/kg bw cisplatin treated. Cis + Mag: Treated with both mangiferin and cisplatin. **(C)** Immunoblot analysis of Nrf-2 on LY294002 and mangiferin pretreated cisplatin exposed NKE cells. LaminB1 was used as a loading control. **(D)** MTT cell viability assay to investigate the effect of PI3K inhibition on the cytotoxic effect of cisplatin on NKE cells. Control: untreated cells; Cisplatin: cells were exposed to 25 μM cisplatin; Cis + Mag: cells were exposed to 25 μM cisplatin and pre-treated with 20 μM mangiferin. Cis+ Mag+ I: cells were exposed to 25 μM cisplatin and pre-treated with 10 μM LY2492002. Each column represents mean ± SD, *n* = 3. “^∗^” Represents the significant difference with the control cells (^∗^*P* < 0.05). “#” Represents significant difference with the mangiferin pretreated cisplatin exposed cells (#*P* < 0.05).

Administration of 10 mg/kg bw cisplatin weekly once for 3 weeks significantly altered the expression of Bax and Bcl2. This subsequently elevated the release of cytochrome C in cytosol. Increased expression of cleaved caspase 3, calpain and cleaved caspase 12 in the kidney tissue of the cisplatin administered animals was observed (**Figure 13A**). In mangiferin and cisplatin co-exposed animals, mangiferin was found to decrease the expression levels of different proteins mentioned above. In addition, mangiferin could significantly induce the expression level of Nrf-2 in the nuclear fraction and SOD-2 and HO-1 in cytosolic fraction of the renal tissue. In the upstream of Nrf-2, it was found that mangiferin pretreatment can significantly elevate the expression of p-PI3K (**Figure 13B**). This result was further confirmed by using LY294002 (a potent inhibitor of PI3K) (Pal et al., 2014). Pretreatment of LY294002 on NKE cells could significantly downregulate the expression of Nrf-2 (**Figure 13C**). Again, by MTT cell viability assay, it was further confirmed that PI3K inhibitor can significantly reverse the protective action of mangiferin (**Figure 13D**).

### Synergistic Anticancer Activity of Cisplatin and Mangiferin *in Vitro* and *in Vivo*

**FIGURE 14:**
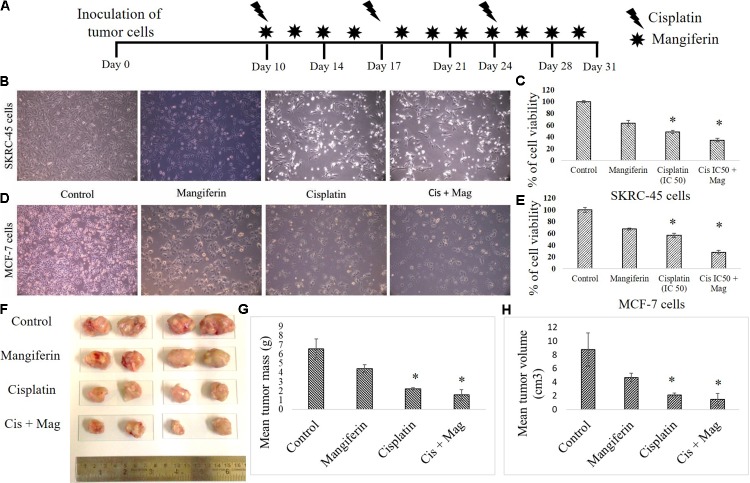
Effect of combined administration of cisplatin and mangiferin on cancer cells and *in vivo* tumors. **(A)** *In vivo* experimental protocol of cisplatin and mangiferin administration. **(B)** Microscopic analysis (100X) on the morphology of the SKRC-45 cells **(C)** effect on cell viability of SKRC-45 cells. **(D)** Microscopic analysis (100X) on the morphology of the MCF-7 cells **(E)** effect on cell viability of MCF-7 cells. Control: untreated cells; Mangiferin: cells were exposed to 20 μM mangiferin; Cisplatin: cells were exposed to 25 μM cisplatin; Cis + Mag: cells were exposed to 25 μM cisplatin and 20 μM mangiferin. **(F)** Tumor photographs dissected out from the experimental animals. **(G)** Mean tumor mass. **(H)** Mean tumor volume. Control: vehicle treated cells. Mag: 20 mg/kg bw mangiferin treated. Cis: 10 mg/kg bw cisplatin treated. Cis + Mag: Treated with both mangiferin and cisplatin. Each column represents mean ± SD, *n* = 4. “^∗^” Represents the significant difference with the vehicle treated animals (^∗^*P* < 0.05). “#” Represents significant difference with the cisplatin administered animals (^#^*P*< 0.05).

To investigate the anticancer efficacy of cisplatin in presence of mangiferin, different *in vitro* and *in vivo* experiments were carried out. It was observed that when mangiferin was pre-exposed to cancer cells (SKRC-45 and MCF-7) prior to cisplatin exposure, a synergistic cytotoxic effect was observed in microscopic studies (**Figures 14B,D**) compared to the cells exposed to mangiferin and cisplatin exclusively. These observations were validated by using MTT cell viability assay. A significant decrease in the cell viability was observed in mangiferin and cisplatin co-exposed cells (34.5% in SKRC-45 cells and 28.04% in MCF-7 cells) compared to the cells exposed to mangiferin (63.56% in SKRC-45 cells and 67.42% in MCF-7 cells) and cisplatin (48.56% in SKRC-45 cells and 56.88% in MCF-7 cells) exclusively (**Figures 14C,E**).

In an *in vivo* experiment with xenograft tumor (EAC cells) bearing experimental animals (**Figure 14A**), mangiferin and cisplatin showed anticancer effect by lowering the mean tumor volume and mean tumor mass compared to animals treated with mangiferin and cisplatin exclusively (**Figures 14F–H**).

## Discussion

In the present study it has been comprehensively demonstrated that (a) Cisplatin can cause severe nephrotoxicity in experimental animals at a dose of 10 mg/kg bw when administered weekly once for 3 weeks. (b) Cisplatin can elevate the level of intracellular ROS in the renal tissue and cause mitochondrial dysfunction. (c) Cisplatin can significantly upregulate different proinflammatory and proapoptotic signaling cascades in the renal tissue. (d) Simultaneous administration of 20 mg/kg bw of mangiferin in alternate days for 3 weeks can effectively ameliorate cisplatin induced organ dysfunction. (e) Mangiferin can upregulate different pro-survival molecules in cisplatin induced pathophysiological state. (f) Along with reno protective effects, mangiferin also exhibits synergistic anticancer activity with cisplatin.

In the last decade, researchers around the globe have primarily focused on identifying novel bioactive molecules from the plant source as there are severe side effects of the chemically synthesized drugs (Carocho and Ferreira, 2013). Amongst different classes of plant bioactive molecules, xanthonoids have gained special importance due to their bioavailability, metabolic stability and numerous prophylactic activities. Mangiferin, a naturally occurring polyphenolic compound, exhibiting anti-oxidant, anti-inflammatory, anti-diabetic and anti-tumor effects, is one of the most potent bioactive xanthonoid molecules identified till date (Benard and Chi, 2015; Fomenko and Chi, 2016). It can be easily extracted and purified from the bark of the mango plant (Dutta et al., 2017). Most interestingly this bioactive molecule has the potential to regulate the expression of different transcription factors (NF-κB, Nrf-2), which can be attributed to its function in regulating cell survival, cell cycle and apoptotic pathways (Gold-Smith et al., 2016; Takeda et al., 2016).

When a free radical is generated in the cellular metabolic process, it is very unstable and can readily react with intracellular biomolecules and can further give rise to other unstable reactive free radicals or a non-reactive stable molecule. Therefore, it is a chain reaction that starts inside a cell to generate free radicals. Under normal physiological conditions, a well-regulated cellular defense mechanism exists to metabolize the free radicals properly and to prevent the accumulation of free radicals in intracellular compartments. However, if there is an excessive generation of free radicals inside the cell, the intracellular enzymatic and non-enzymatic antioxidant machinery fail to metabolize them (free radicals) properly. Hence free radicals get accumulated and lead to oxidative stress. Free radical scavengers are found to be very beneficial in such stressed conditions. In various oxidative stress mediated renal pathophysiology, it has been found that natural antioxidants are effective in restoring the cellular antioxidant defense mechanism (Rafieian-Kopaei et al., 2013). Previous results also suggest that quercetin administration has prophylactic effect against cisplatin induced oxidative stress in renal tissue. Oral administration of mangiferin has been found to ameliorate oxidative stress induced major organ dysfunctions by escalating the intracellular level of GSH and the activities of other chief antioxidant enzymes like SOD, CAT, GST, GR and GPx (Sies, 2015; Zhang and Tsao, 2016; Poprac et al., 2017). In the present study, cisplatin exposure was found to diminish the intracellular antioxidant mechanism both *in vitro* and *in vivo* and simultaneous administration of mangiferin was found to be effective in attenuating the deleterious effects of ROS by replenishing the level of GSH and other antioxidant enzymes.

**FIGURE 15:**
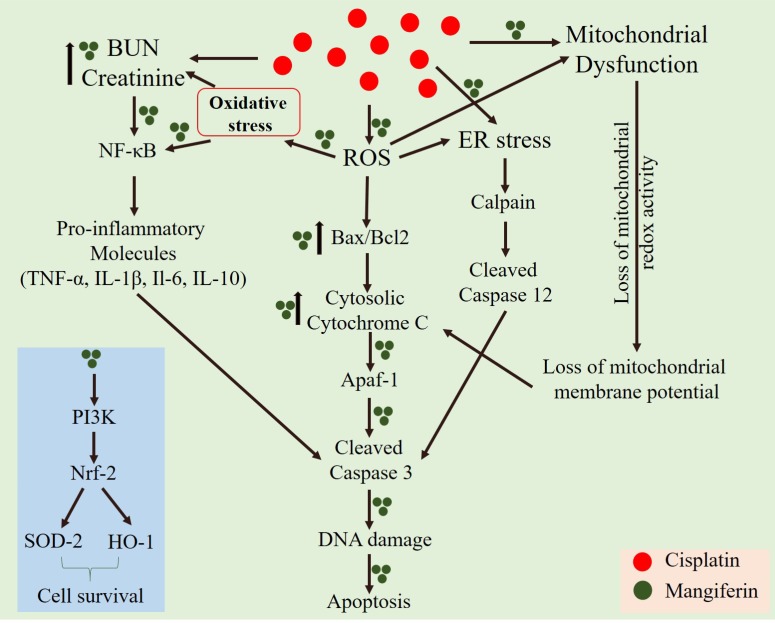
A schematic representation of the mechanism of cytotoxicity induced by cisplatin at a dose of 10 mg/kg bw weekly once for 3 weeks and the ameliorative effect of mangiferin when administered at a dose of 20 mg/kg bw on every alternative day for 3 weeks.

Accumulation of intracellular ROS has a direct relationship with mitochondrial dysfunction, induction of pro-inflammatory cytokines through the nuclear translocation of NF-κB and different proapoptotic cellular signaling cascades (Manna et al., 2008b; Wang et al., 2014; Luo et al., 2015). In our *in vitro* and *in vivo* cytotoxicity model, it was observed that cisplatin could induce mitochondrial dysfunction and activate different pro-inflammatory cytokines. Our *in vivo* study indicates that cisplatin administration in the experimental animals caused nuclear translocation of NF-κB. The RT-PCR results also indicated the upregulation of several proinflammatory cytokines viz., TNF-α, IL-1β, IL-6 and IL-10. Simultaneous administration of mangiferin was found to be effective in preventing the nuclear translocation of NF-κB and attenuate the elevated expression of several pro-inflammatory cytokines elicited by cisplatin. It was also observed that cisplatin can effectively activate caspase dependent apoptotic pathway mediated by mitochondrial dysfunction and induction of ER stress. It was observed that mangiferin significantly restores the altered Bax/Bcl-2 ratio by attenuating the mitochondrial dysfunction and causing release of cytochrome C in the cytosol from the mitochondria. The immunoblot analyses in this study also indicated that cisplatin administration significantly upregulated the two chief regulators of ER stress, calpain and caspase 12 compared to the control animals. Reports suggest that calpain facilitate the cleavage of pro caspase 12 and gives rise to activated caspase 12 (Martinez et al., 2010; Huang et al., 2014; Pal et al., 2015a). This activated caspase 12 translocates to the cytoplasm and activates procaspase 3 to induce apoptosis. In line with the previous observations, mangiferin was found to regulate the expression of calpain and thereby prevent the activation of caspase dependent apoptotic pathway compared to the cisplatin administered animals. Moreover, simultaneous administration of mangiferin was effective in upregulating several other pro-survival molecules (Das et al., 2011; Saha et al., 2016a). It was observed that mangiferin administration in presence of cisplatin can upregulate the expression of Nrf-2 in the renal tissue via the activation of PI3K. Previous reports also showed that mangiferin exposure only alters the expression of p-PI3K without altering the expression of total PI3K (Qi et al., 2017; Zou et al., 2017). Besides, p-PI3K can directly upregulate the expression of Nrf-2 and can significantly promote cell survival (Saha et al., 2016c). Nrf-2 can also upregulate the expression of SOD-2 and HO-1 in the renal tissue compared to cisplatin administered animals. This could be a possible explanation behind the prophylactic role of mangiferin against cisplatin induced nephrotoxicity (Kavitha et al., 2013; Mahmoud-Awny et al., 2015; Saha et al., 2018). A schematic representation of the mechanism of cytotoxicity induced by cisplatin and the ameliorative effect of mangiferin has been given in **Figure 15**.

Most interestingly, though mangiferin attenuates the cytotoxic effect of cisplatin in the renal tissue, it acts synergistically when administered simultaneously with cisplatin in the xenograft tumor bearing mice (EAC cells). This differential effect of mangiferin was also observed in two different secondary cell lines, SKRC-45 and MCF-7. SKRC-45 cells are the metastatic renal tubular cancer cells and MCF-7 is a well-known model to study breast cancer. The differential effect of mangiferin in renal and tumor tissue may be because of the varied nature of cells and structural organization of the two tissues (Sánchez-González et al., 2017).

## Conclusion

Overall the present study suggests that the polyphenolic bioactive natural compound mangiferin, which is abundantly found in the bark of the mango tree, is highly effective against cisplatin induced nephrotoxicity without affecting its (cisplatin) beneficial effect on the tumor tissue. Rather, simultaneous administration of mangiferin showed a synergistic anti-tumor activity with cisplatin. The prophylactic nature of mangiferin can be attributed to its antioxidant and anti-inflammatory properties. It can therefore be concluded that mangiferin can be considered as a promising drug candidate to be used in combination with cisplatin to ameliorate the nephrotoxic effects of cisplatin and obtain a synergistic anticancer activity.

## Author Contributions

PS, SS, SD, and PCS were actively involved in designing the experiments. PS, SS, and SD performed the experiments. PS, SS, and PCS analyzed the data and prepared the manuscript.

## Conflict of Interest Statement

The authors declare that the research was conducted in the absence of any commercial or financial relationships that could be construed as a potential conflict of interest.
